# Overweight is not predictive of reduced effectiveness of orthosis treatment in Adolescent Idiopathic Scoliosis: results from a retrospective cohort

**DOI:** 10.1186/1748-7161-8-S2-O61

**Published:** 2013-09-18

**Authors:** Fabio Zaina, Sabrina Donzelli, Monia Lusini, Salvatore Minnella, Luca Vismara, Paolo Capodaglio, Stefano Negrini

**Affiliations:** 1 ISICO Italian Scientific Spine Institute, Milan, Italy

## Background

A previous study reported that being overweight is predictive of decreased effectiveness of bracing for patients diagnosed with Adolescent Idiopathic Scoliosis (AIS), with the risk of worsening three times higher in overweight patients than in normal-weight scoliosis patients. 

## Purpose

The goal of this study was to verify if body mass index (BMI) and, specifically, obesity, are predictive for the effectiveness of bracing in a cohort of AIS patients. 

## Methods

Design: retrospective cohort study. Population: 351 AIS patients (306 females), age 10-15 years at first assessment (average 12.9±1.4), worst curve 35.6±11.4° Cobb, ATR 11±4.3°, median Risser 2, BMI 19.7±3. All subjects included were prescribed a brace treatment at first visit (initially 18-23 hours per day) accompanied by Scoliosis Physiotherapic Exercise according to the Scientific Exercises Approach to Scoliosis (SEAS) protocol. After the treatment, all patients were evaluated again and changes were analysed. Outcome: n° of patients improved/worsened (defined as patients with a change of more than 5° Cobb) or stable, average change of Cobb angle. Statistical analysis: a stepwise linear regression was used to look for the effect of BMI as a predictor of result. A chi-square test and a logistic were used for the category of overweight patients (BMI³85° percentile). During the statistical analysis, we controlled for possible confounders. 

## Results

The chi-square test showed similar results in overweight and normal weight 44% vs. 52% improved, 52% vs. 41% stable and 3% vs. 7% worsened, respectively. We found BMI to be poorly correlated with final results. Adjusting for confounders did not change this poor correlation, and the predictive model explained about 10% of the result. 

## Conclusions and discussion

We found similar results in overweight and normal weight subjects treated with a brace for AIS. This is in contrast with a previous study that suggested the overweight condition to be a contraindication for brace treatment due to poor results. It is possible that these differences in results depend on the management of the treatment, which in our study followed the indication of the SOSORT guidelines. 

Further studies are needed to confirm our results in different settings and populations.
